# Serum Cytokine Profile by ELISA in Patients with Echinococcal Cysts of the Liver: A Stage-Specific Approach to Assess Their Biological Activity

**DOI:** 10.1155/2012/483935

**Published:** 2012-02-07

**Authors:** Luca Piccoli, Valeria Meroni, Francesca Genco, Francesca Tamarozzi, Carmine Tinelli, Carlo Filice, Enrico Brunetti

**Affiliations:** ^1^Department of Infectious Diseases, IRCCS San Matteo Hospital Foundation, University of Pavia, and WHO Collaborating Centre for Clinical Management of Cystic Echinococcosis, Pavia 27100, Italy; ^2^Laboratory of Parasitology, Department of Infectious Diseases, IRCCS San Matteo Hospital Foundation, University of Pavia, Pavia 27100, Italy; ^3^Clinical Epidemiology and Biometric Unit, IRCCS San Matteo Hospital Foundation, Pavia 27100, Italy

## Abstract

To investigate the usefulness of serum cytokine dosage in the clinical management of cystic echinococcosis (CE), we analyzed serum levels of Th1 and Th2 cytokines in patients with hepatic CE in different cyst stages, CE1-2 (active), CE3a-3b (transitional), and CE4-5 (inactive). *Ex vivo* assessment of Th1 (IFN-**γ**) and Th2 (IL-4, IL-13, and IL-10) cytokines in sera was carried out using ELISA. IL-10 was undetectable in all serum samples of patients and controls, while a few sera contained measurable amounts of IFN-**γ**, IL-4, and IL-13. No statistically significant difference was found between the percentages of positive samples for each cytokine and the different groups analyzed (patients/controls, stage, number, location, and size of the cyst, serology, and sex of patients), with the exception of the association of IL-4 and IL-13 with the cyst stage. Overall, this investigation showed many limits of serum cytokine dosage as a marker of biological activity of echinococcal cysts. Because of low sensitivity and lack of specificity of this test, we believe that other ways to evaluate *ex vivo* biological activity of the cysts should be explored.

## 1. Introduction

Cystic echinococcosis (CE) is a chronic infection caused by the tapeworm *Echinococcus granulosus*. In humans, the larval stage of the parasite can develop and form cysts in almost any organ, especially the liver and the lungs [1]. Diagnosis and clinical decision making of CE are currently based on imaging techniques, mainly ultrasound (US) and, to a lesser extent, on serological techniques.

To date, serology is not standardized, and specific antibodies may persist for a long time, even after complete surgical removal of the cyst [2, 3]. Furthermore, biological activity of transitional cysts does not always match the US appearance of the echinococcal cyst [4]. As a consequence, cyst progression towards either inactivation or chronicization can be assessed only by changes in US appearance of the cyst and, to a lesser extent, by variation in antibody titers, which is demonstrated only over long-term followup, thus making serology less useful than US [5, 6].

To evaluate the biological activity of the cyst as a tool for clinical decision making, in the last decade several studies have tried to look for markers of the immune response against *Echinococcus granulosus.* Some *in vitro* studies, investigating cytokine production from peripheral blood mononuclear cells of CE patients, demonstrated the presence of both Th1 and Th2 response against the parasite. During chronicization of the infection, the more permissive Th2 response predominates in patients with active (or not cured) cysts over the parasite-damaging Th1 response which, on the contrary, is more active in patients with inactive (or cured) cysts [7–10].

Other *ex vivo *studies, which detected cytokines in sera of CE patients, confirmed the association between cytokine production and outcome of the disease. Rigano et al. reported a higher serum level of Interleukin-4 (IL-4) and IL-10 in patients who did not respond to therapy compared to those who responded; Bayraktar et al. showed higher concentrations of IL-2, IL-4, and IL-10 in CE patients before treatment compared to those who were treated and to healthy controls; Mezioug and Touil-Boukoffa observed the coexistence of elevated levels of Interferon-*γ* (IFN-*γ*), IL-12, IL-16, IL-18, IL-4, IL-5, IL-10, and IL-13 in most sera of CE patients compared to healthy controls [11–13].

Although these studies showed an association between serum cytokine concentrations and active CE, in a recent study we could not confirm such association for all cytokines, as only a subgroup of CE patients with transitional cysts showed increased IL-4 levels compared to other subgroups and negative controls [14]. Additionally, these studies did not stratify patients according to the different cystic stages at US, which have been shown to correlate well with the biological activity of the cysts, with the exception of transitional stages [4, 15].

In this study, we analyzed serum levels of IFN-*γ*, IL-4, IL-13, and IL-10 in patients with hepatic CE in different US stages, to evaluate *ex vivo* the association of cytokine production and the stage of the infection. A second aim of the study was to assess whether serum cytokine dosage, which could be easily implemented in a clinical setting, can reliably assess the biological activity of CE cysts.

## 2. Materials and Methods

### 2.1. Subjects and Serum Samples

Serum samples were obtained from 53 CE patients seen at the Department of Infectious Diseases of the IRCCS San Matteo Hospital Foundation in Pavia, Italy, and from 20 healthy controls. The study protocol was approved by the ethical committee of our institution, and all subjects gave their informed written consent. Diagnosis of CE was made by ultrasound and serological assays, and patients were selected according to these inclusion criteria: (i) presence of at least one CE cyst localized to the liver, (ii) no previous surgery for CE, and (iii) no albendazole (ABZ) treatment or ABZ discontinuation at least 24 months before the time of serum collection. The control group was constituted by people for whom CE could be excluded by both abdominal US and serological assays. Serum samples were collected during a period of two years (from March 2009 to March 2011) and stored at −80°C until assayed.

### 2.2. Ultrasound

**Figure 1:**
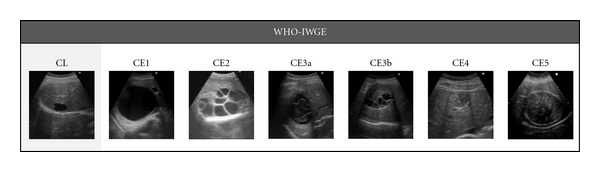
WHO-IWGE ultrasound classification of echinococcal cysts.

All patients and controls were examined by a clinician with long-standing experience in US and clinical management of CE (EB) using a commercially available US scanner with 3.5–5 MHz convex probes (Aloka ProSound ALPHA 10, Tokyo, Japan). For each patient, number, stage, size and location of the cysts were reported. Cysts were classified according to the World Health Organization Informal Working Group on Echinococcosis (WHO-IWGE) standardized US classification for CE [16] (Figure 1) as CE1 and CE2 (active), CE3 (transitional), and CE4 and CE5 (inactive). Transitional CE3 cysts were further divided into 2 subgroups, CE3a and CE3b, based on their difference in response to nonsurgical treatments and biological activity [17]. Patients having multiple cysts were classified according to the more active stage, in accordance with the results of Hosch et al. [4]. Cyst size was reported as small, medium or large, if the greatest cyst diameter was lower than 5 cm, between 5 and 10 cm, or greater than 10 cm, respectively. Cyst location in the liver was classified as being in the right, left, or fourth segment.

### 2.3. Serology

All patients and controls were tested for anti-*Echinococcus* antibodies by IgG enzyme-linked immunosorbent assay (ELISA, Cypress Diagnostic, Langdorp, Belgium) and indirect hemagglutination (IHA, Cellognost Echinococcosis; Dade Behring, Newark, USA) by the Laboratory of Parasitology of our hospital. All controls and patients visited were tested for IgG western blot (*Echinococcus* western blot IgG, LDBIO, Lyon, France) during their first visit at our clinic. ELISA was considered positive if optical density was greater than 1.1, while IHA tested positive for dilution greater than 1/64. Serology was defined as either positive or negative, if both ELISA and IHA tested either positive or negative respectively, and, if necessary, were confirmed by WB; serology was defined as doubtful if one test was not congruent with the other one(s).

### 2.4. Cytokine Assays

Serum concentrations of IFN-*γ*, IL-4, IL-13, and IL-10 were determined by ELISA commercial kits (human IFN-*γ*, IL-4, and IL-10 high-sensitivity ELISA kits and human IL-13 ELISA kit, Gen-Probe Diaclone, France) according to the manufacturer's instructions. All tests were performed in duplicate. The ranges of the sensitivity standard curve of the ELISA kits were 0.78–25 pg/mL for IFN-*γ*, 0.31–10 pg/mL for IL-4, 3.12–100 pg/mL for IL-13, and 1.56–50 pg/mL for IL-10.

### 2.5. Statistical Analysis

Differences in percentages of patients and controls with detectable levels of each cytokine were assessed by Fisher's exact test. The same test was applied to assess any associations between cytokines and stage, number, location, and size of the cysts, serology, and sex of patients. A *P* value of less than 0.05 was considered statistically significant, and all tests were two sided. Data analysis was performed with the STATA statistical package (Ver. 10.0, 2009, Stata Corporation, College Station, TX, USA).

## 3. Results

The results are summarized in Table 1.

### 3.1. Subjects

This study included 73 subjects, 53 of whom were patients with liver CE cysts in different US stages, while 20 were healthy controls; in the control group CE could be excluded by both abdominal US and serological assays that tested negative. Of the 53 patients, 25 (47.2%) were males and 28 (52.8%) were females. Forty-six (86.8%) harbored one cyst each, while 7 (13.2%) harbored two cysts each. Five had active CE1-CE2 cysts (9.5%), 8 had transitional CE3a cysts (15.1%), 20 had transitional CE3b cysts (37.7%), and 20 had inactive CE4-CE5 cysts (37.7%). Forty-one patients (77.4%) had their cysts located in the right segments of the liver, 5 (9.4%) in the left segments, and 7 (13.2%) in the fourth segment. Seventeen patients (32.1%) had small-sized cysts, 27 (50.9%) had medium-sized cysts, and 9 (17.0%) had large-sized cysts. Serology was positive in 29 patients (54.7%), negative in 19 patients (35.9%), and doubtful in 5 (9.4%) patients.

### 3.2. Cytokine Dosages and Associations

IL-10 was undetectable in all 73 serum samples of patients and controls, while a few sera contained measurable amounts of IFN-*γ*, IL-4, and IL-13; percentages of positive samples were 17.8%, 16.4%, and 13.7%, respectively. No statistically significant difference was found between the percentages of positive samples for each cytokine and the different groups analyzed (patients/controls, stage, number, location and size of the cyst, serology, and sex of patients), with the exception of the association of IL-4 and IL-13 with the cyst stage (*P* = 0.010 and *P* = 0.033, resp.). This was likely due to higher percentages of positive samples for IL-4 (50%) and IL-13 (37.5%) in the CE3a-stage group compared to the other cystic stages. The low number of patients in each group prevented us from evaluating any intergroup statistical differences.

## 4. Discussion

To date, ultrasound and serology are very useful to diagnose and monitor the evolution of cystic echinococcosis, but a marker of activity of the cyst is still lacking. Therefore, clinical decision making may be challenging, in particular for those cases that tend to relapse after an initial successful treatment [1, 17].

It is well known that CE patients generate both Th1 and Th2 immune responses, which skew towards the Th2 arm in the chronic phase [7, 18–22]. In this study, we evaluated the presence of Th1 (IFN-*γ*) and Th2 cytokines (IL-4, IL-13, and IL-10) in the sera of patients with different cystic stages according to the WHO-IWGE classification of echinococcal cysts [17]. Unlike previous publications [12, 13, 20], our results show that there is no difference in the presence of IFN-*γ*, IL-4, and IL-13 in sera of patients compared to negative controls. This is possibly because cytokines are not specific for a particular disease, as they are produced in every Th1- or Th2-mediated inflammatory process. Furthermore, Diaz et al. [23] recently reviewed the structure of the laminated layer of the echinococcal cyst and pointed out its possible role in downregulating both Th1 and Th2 response, thus allowing parasite survival. This aspect could explain the low percentage of positive samples containing detectable amounts of IFN-*γ*, IL-4, and IL-13. Interestingly, IL-10 could not be detected in any samples analyzed. This could be due to a sensitivity limit of the ELISA kit employed. Furthermore, in the literature reviewed by Diaz et al. [23], IL-10 is expressed by leukocytes in infected hosts, especially in the immediate vicinity of the parasite. This aspect could explain the difficulty in dosing IL-10 directly in serum samples.

The comparison of the percentages of positive samples for each cytokine and the different groups analyzed (patients/controls, stage, number, location, and size of the cyst, serology, and sex of patients) did not show any statistically significant differences, with the exception of the association of IL-4 and IL-13 with the cyst stage. This result indicates that the percentage of positive samples for both cytokines is not equal between the different cyst stages, perhaps because of the higher percentage of positive samples for IL-4 and IL-13 in the CE3a-stage group compared to the other cystic stages. Furthermore, these results do not confirm a previous finding by our research group, which showed that the percentage of IL-4-positive samples was higher in CE3b patients compared to other groups [14]. A limitation of these studies is the small sample size which is not sufficient to evaluate any intergroup statistical differences.

Cytokines are immune regulators, which are produced by many cells and have short half-lives. Therefore, their measurement in serum is difficult, and results of tests can be influenced by several variables, such as sample collection protocols (sample handling, processing, and storage) and patient behaviors prior to collection (dietary habits, food ingestion, physical activity, and stress) [24].

Overall, this investigation showed many limits of serum cytokine dosage as a marker of biological activity of echinococcal cysts. Because of low sensitivity and lack of specificity of this test, we believe that other ways to evaluate *ex vivo* biological activity of the cysts should be explored.

## Figures and Tables

**Table 1:** Distribution of positive samples for IFN-*γ*, IL-4, and IL-13 in the different groups analyzed (patients/controls, stage, number, location, and size of the cyst, serology, and sex of patients). *Difference statistically significant (*P* value < 0.05); the remaining differences are not significant.

Group	Total	IFN-*γ* positive		IL-4 positive		IL-13 positive	
	no.	no.	%	no.	%	no.	%
Total subjects	73	13	17.8	12	16.4	10	13.7
Controls	20	5	25	5	25	2	10
Patients	53	8	15.1	7	13.2	8	15.1

Cyst stage	
CE1-2	5	0	0	1	20*	1	20*
CE3a	8	3	37.5	4	50	3	37.5
CE3b	20	4	20	1	5	0	0
CE4-5	20	1	5	1	5	4	20

Cyst number	
1	46	7	15.2	6	13	6	13
2	7	1	14.3	1	14.3	2	28.6

Cyst liver location	
Right segment	41	6	14.6	7	17.1	6	14.6
Left segment	5	1	20	0	0	0	0
IV segment	7	1	14.3	0	0	2	28.6

Cyst size	
Small	17	1	5.9	2	11.8	1	5.9
Medium	27	5	18.5	4	14.8	6	22.2
Large	9	2	22.2	1	11.1	1	11.1

Serology	
Positive	29	6	20.7	6	20.7	4	13.8
Negative	19	2	10.5	0	0	2	10.5
Doubtful	5	0	0	1	20	2	40

Sex	
Male	25	4	16	5	20	4	16
Female	28	4	14.3	2	7.1	4	14.3
